# Chitosan-Based Dressing Materials for Burn Wound Healing

**DOI:** 10.3390/polym17121647

**Published:** 2025-06-13

**Authors:** Shiyu Li, Wenlong Pan, Ming Zhang, Kailu Song, Ziqian Zhou, Qilong Zhao, Guang-Zhao Li, Chongyu Zhu

**Affiliations:** 1 College of Chemistry and Chemical Engineering, Donghua University, Shanghai 201620, China; 2 Key Laboratory of Materials and Surface Technology (Ministry of Education), School of Materials Science and Engineering, Xihua University, Chengdu 610039, China

**Keywords:** chitosan, burn wound, dressing forms, antimicrobial activity, antioxidative activity, tissue engineering

## Abstract

The treatment of burn injuries remains a significant global challenge. Although conventional cellulose-based dressings are still the dominant clinical choice, chitosan-based burn wound dressing materials have emerged as a promising alternative due to their unique physicochemical properties and biocompatibility. In this mini-review, we aim to provide a summary of recent advances in chitosan-based dressing materials and highlight their advantages in the treatment of burn wounds. Specifically, we first outline the chemical structure and synthesis methods of chitosan and its derivatives. Subsequently, various forms of chitosan-based dressings are introduced, with a particular focus on hydrogels and micro/nanofibers dressings, along with an overview of their preparation methods. Considering the microenvironment of the burn wound site, we then summarize the design principles and clinical efficacy of chitosan-based dressings with antimicrobial and/or antioxidative activity. Additionally, the applications of chitosan dressings in tissue engineering for burn treatment are also discussed, including growth factor delivery, gene therapy, and stem cell-based treatments. Finally, we examine the main challenges of chitosan-based dressing materials and the potential future directions. Through this mini-review, we expect to provide new perspectives for the development of wound dressings for burn care.

## 1. Burn Wound and Healing Materials

Burns or burn wounds are injuries to the skin caused by various factors, including heat, electricity, chemicals, and radiation [1]. Depending on the severity of the skin damage, burns are typically classified into four degrees [2]. A first-degree burn affects the superficial layers, primarily involving the epidermis. Second-degree burns involve both the epidermis and dermis. Based on the depth of dermal damage, second-degree burns can be further classified into superficial partial-thickness burns and deep partial-thickness burns. A third-degree burn affects the full thickness of the skin, including the subcutaneous fat. A fourth-degree burn extends beyond the skin into the underlying muscles, tendons, and even bones [3]. While superficial burn wounds that only affect the epidermal layer of the skin often heal rapidly without medical intervention, deep burns or those covering a large total body surface area (TBSA) can pose a significant life-threatening risk and may require whole blood replacement and surgical intervention [4,5]. Consequently, the treatment of burn wounds remains a major concern in modern society.

Burn wound healing typically consists of several overlapping and continuous phases: the hemostasis/inflammatory, cellular proliferation and matrix remodeling. Compared to other types of wounds, burn wounds often cause vascular endothelial hyperpermeability, leading to the extravasation of plasma components (e.g., water, small molecules, and proteins) into interstitial space and the subsequent formation of a large amounts of exudates [6]. The nutrient-laden exudates along with necrotic tissues provide microenvironment for bacterial growth, increasing the risk of infection. Moreover, extensive or deep burns wound healing slowly and are prone to hypertrophic scarring and contractures, which affects the appearance and functions of the burnt area. Therefore, it is crucial to strengthen burn wound management to prevent infection, promote tissue regeneration and vascular reconstruction [7].

To address the challenges in burn wound treatment, the development of effective burn wound dressings is critical. While traditional cellulose-based dressings like gauze [8] have proven successful, other bio-derived polymers like sodium alginate [9], silk fibroin [10], collagen [11], and particularly chitosan and its derivatives, have emerged as highly promising alternatives. Among all, burn wound dressings formulated with chitosan and its derivatives stand out due to their cost-effective raw materials, while offering excellent biocompatibility and biodegradability. These features allow them suitable as burn wound dressings to conform the wound healing process and minimizing the risk of adverse reactions or secondary trauma upon removal [12]. Moreover, they possess intrinsic antimicrobial properties, providing a “clean” wound healing environment against infections [13]. Additionally, their hydrophilic nature allows them to absorb wound exudate and maintain a moist wound bed, which supports optimal healing conditions [14]. These attributes, combined with their potential to stimulate cell growth and tissue regeneration, make chitosan and its derivatives be valuable materials in advanced burn wound dressing formulations [15,16,17].

In this review, we will provide an overview of the recent advancements in chitosan-based dressing materials for burn wound healing in the past decade. We will first introduce the fundamental of chitosan and its derivatives, including their synthesis and chemical/physical properties. Next, we will explore the two major types of burn wound dressing forms (hydrogels and micro/nanofibers), highlighting their structural features and the advantages of each form. Finally, we will discuss the functional aspects of chitosan-based dressings in burn care, focusing on their ability to prevent infections, reduce oxidative stress, and their applications in tissue engineering. Through this mini-review, we aim to offer a deeper insight into the potential and advantages of utilizing chitosan and its derivatives for the management of burn wounds.

## 2. Chitosan and Its Derivatives for Burn Wound Dressings

Chitosan is a cationic polysaccharide obtained through the deacetylation of chitin, whose unique biological advantages original from its structural properties. Its parent structure, chitin, is found in crustacean exoskeletons [18], insect cuticles, and fungal cell walls [19]. Chitin possesses great biocompatibility, owning to its structure which is similar to extracellular glycosaminoglycans and its enzymatic degradation into non-toxic com-ponents. When chemically converted to chitosan, this biopolymer acquires protonatable amino groups that confer pH-dependent cationic charges at physiological conditions. These charged moieties facilitate electrostatic interactions with anionic cell membrane components, enhancing cellular adhesion and proliferation capabilities.

The functional properties of chitosan are collectively determined by three interrelated parameters, including biotic origin, degree of deacetylation, and molecular weight. First, fungal-derived chitosan is safer and less allergenic than crustacean-derived chitosan due to the absence of tropomyosin [20]. Second, the degree of deacetylation, as a core regulatory factor, simultaneously influences the physicochemical characteristics and biological behavior of material. A higher degree of deacetylation enhances crystallinity [21] and thermal stability [22], as well as increases surface charge density by exposing more amino groups. This allows chitosan to improve mechanical properties [23,24] by reinforcing structural integrity and resisting degradation, meanwhile optimizing biological performance by strengthening electrostatic interactions with cells. Finally, differences in molecular weight affect polymer chain entanglement dynamics and degradation rates, providing additional tunability for specific applications.

Chitosan, as a copolymer, is formed by the linkage of N-acetyl-D-glucosamine and D-glucosamine units through β-(1,4)-glycosidic bonds [25]. Notably, the amino groups on these D-glucosamine units make chitosan a unique bio-derived cationic polysaccharide, thereby endowing it with inherent antimicrobial properties [26]. Meanwhile, due to the abundant intermolecular and intramolecular hydrogen bonds within its molecular structure, chitosan displays limited solubility in water and most organic solvents [27,28], which sometimes causes problems during the preparation and application of burn wound dressings [29,30].

To improve the solubility of chitosan, chemical modification has emerged as an effective strategy [31,32]. Through chemical modification, the hydrophilicity/hydrophobicity of the side chains of chitosan is altered and the chain length of chitosan is often shortened, which weakens its intramolecular and intermolecular hydrogen bonding [33]. This, in turn, reduces its crystallinity and improves its water solubility [34]. Generally, the chemical modification of chitosan targets the two functional groups on its D-glucosamine units: the amino group at the C-2 position and the hydroxyl group at the C-6 position owing to their pronounced chemical reactivity [35] (Table 1). For instance, by introducing hydrophilic groups such as carboxyl groups [36], hydroxyl groups, sulfonic acid groups, quaternary ammonium salts, and phosphorylated groups [37], chitosan derivatives with improved water solubility can be obtained. Such examples including commercially available source like carboxymethyl chitosan, glycol chitosan, and hydroxypropyl chitosan. Among them, carboxymethyl chitosan exhibits good solubility in aqueous solutions across wide pH range and demonstrates excellent processing compatibility with other biocompatible macromolecules such as sodium alginate and dextran, making it highly favored in burn wound dressings [38].

In addition to improving solubility, chemical modification of chitosan, particularly at the amino groups in the C-2 position, can impart additional functions such as antioxidative activity. For instance, chitosan derivatives modified with polyphenol structures such as gallic acid [46] and catechol [47] can be manufactured into burn wound dressings with antibacterial and antioxidant properties, promoting wound healing. Furthermore, these polyphenol structures can further interact with other compounds (i.e., MgO [48], Fe(III) ions [49]) to form additional crosslinking, thereby allowing for the fine-tuning of the mechanical strength of the chitosan-based burn wound dressing.

## 3. Forms of Chitosan-Based Burn Wound Dressings

**Figure 1:**
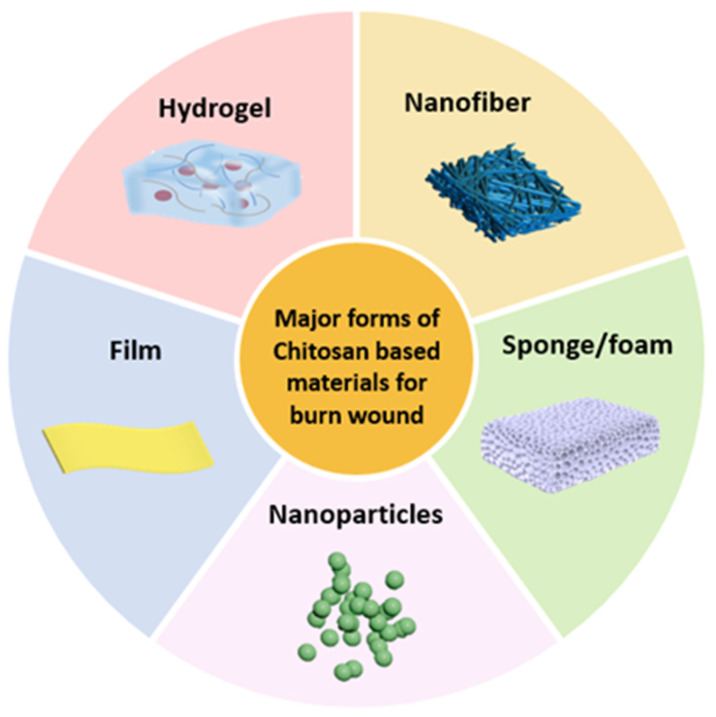
Major forms of chitosan-based wound dressing materials.

To address the diverse needs of burn treatment, various forms of chitosan-based burn wound dressing materials have been developed [50], including films [51], sponges/foams [52], nanoparticles [53], hydrogels [54] and micro/nanofibers [55] (Figure 1). Each form provides different benefits suited to clinical situations. For instance, nanoparticles are typically administered as liquid coatings or through spray-based delivery systems for topical wound application. Alternatively, chitosan-based films function as temporary protective barriers post-debridement, while their sponge/foam variants specialize in exudate absorption through porous architectures. Although all these forms have demonstrated therapeutic potential in burn wound management, chitosan-based hydrogels and micro/nanofibers remain the most extensively researched dressing formats in the past decade [16,56,57]. This is mainly attributed to their superior exudate absorption, improved gas permeability and better mechanical match to the wound areas compared to other forms [58]. More importantly, their porous structures can mimic the natural extracellular matrix, providing a scaffold for cell growth and thereby promoting tissue regeneration [54,55], which is beneficial for deep partial-thickness burn wounds or third-degree burn wounds healing. Therefore, in this section, we will focus on the forms of these two major categories and introduce a brief overview on their preparation methods.

Possessing a three-dimensional network structure, hydrogel exhibits comparable mechanical properties to the skin and holds a large amount of water within its structure [59,60]. Hence, burn wound dressings in the form of hydrogel not only act as a physical barrier for wounds, but also offer effective wound exudate absorption, maintaining a moist environment favorable for wound healing [61]. In addition, hydrogel-based burn wound dressings can offer sustained hydration and localized cooling, reducing pain at the wound area [2,61]. Furthermore, the high porosity of these hydrogels not only facilitates smooth gas exchange between the burn wound and the external environment [62], but also enables them to serve as effective drug carriers [63].

**Figure 2:**
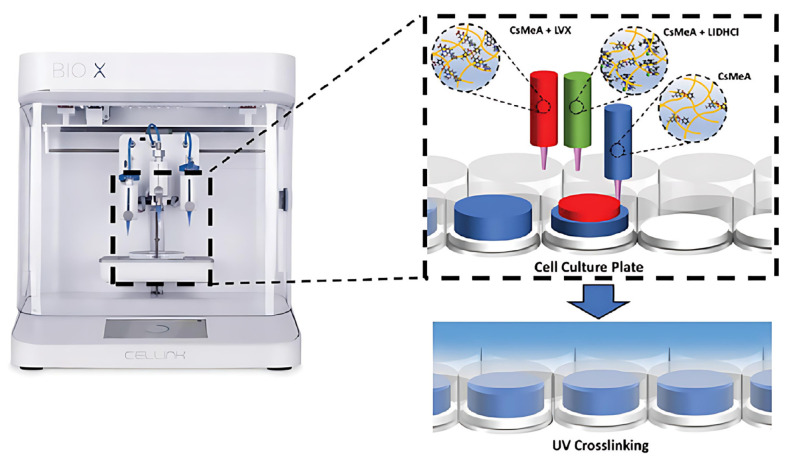
Schematic diagram of manufacturing personalized chitosan methacrylate loaded with lidocaine hydrochloride (LIDHCI) or levofloxacin (LVX) using a multi-nozzle printer. Adapted with permission from Ref. [69]. Copyright © 2021 Wiley-VCH GmbH (Weinheim, Germany).

The preparation of chitosan-based hydrogel wound dressings typically relies on chemical crosslinking reactions targeting the amino groups in chitosan [64,65]. Common crosslinking agents, such as glyoxal [66] and glutaraldehyde [67], react with amino groups to form stable three-dimensional network structures [68]. Alternatively, the amino groups in chitosan can be further modified with photocrosslinkable groups, such as (meth)acrylic anhydrides, which enables the fabrication of tailored hydrogels via digital light processing (DLP)-based three-dimensional (3D) printing. Wang et al. demonstrated this by developing a methacrylated chitosan hydrogel precursor containing a photoinitiator [69]. By further incorporating therapeutic agents like lidocaine hydrochloride (LIDHCI) and levofloxacin (LVX), they developed drug delivery systems on demand for personalized applications such as pain management or infection-resistant burn wound dressings (Figure 2).

**Figure 3:**
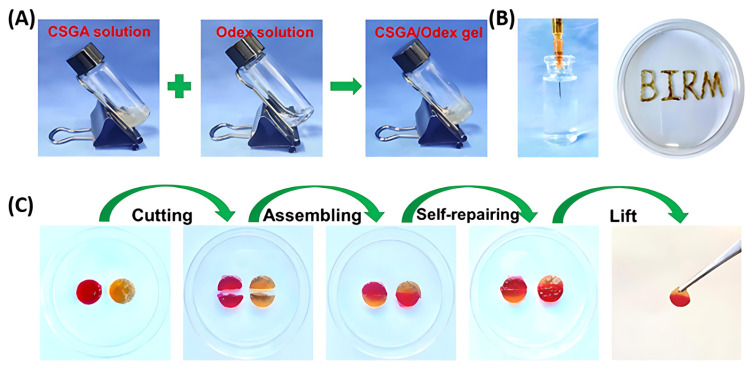
(**A**) The hydrogel by mixing gallic acid-modified chitosan (CSGA) solution and oxidized dextran (ODex) solution. (**B**) CSGA/ODex injectable hydrogel. (**C**) The self-healing property of CSGA/ODex hydrogels. Adapted with permission from Ref. [72]. Copyright © 2023 Published by Elsevier Ltd. (Amsterdam, The Netherlands).

It is noteworthy that the crosslinkers do not necessarily need to form stable crosslinking points to produce hydrogels. Cross-linking can also be formed by dynamic bonds [70] or weaker interactions such as hydrogen bonds, electrostatic attraction, or hydrophobicity [71]. For example, Shen et al. developed a hydrogel by reacting the amino groups of a chitosan derivative (gallic acid-modified chitosan, CSGA) with the aldehyde groups of oxidized dextran via dynamic Schiff base reaction [72] (Figure 3A). Benefiting from the reversibility of Schiff base linkages, this formed hydrogel not only exhibits self-healing properties, but also can be continuously extruded through needles (Figure 3B,C), filling in irregular wound areas and promote in situ regeneration.

**Figure 4:**
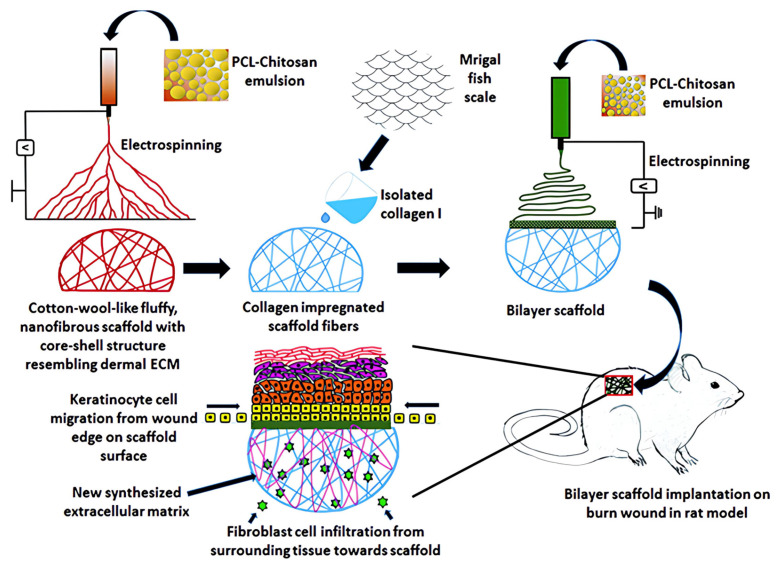
Schematic diagram representing development of a bilayer electrospun dressing and its application to healing burn wound in a rat model. Reprinted with permission from Ref. [75]. Copyright © 2013 Royal Society of Chemistry (London, UK).

Chitosan-based micro/nanofibers, characterized by their high porosity [55], represent another promising candidate for burn wound treatment due to their excellent gas permeability, effective hemostasis as well as exudate absorption capabilities. Through electrospinning technology, chitosan-based micro/nanofibers can be engineered into core–shell structures [73] or mesoporous structures [74] and subsequently processed into functional dressings. Additionally, this technology facilitates the preparation of chitosan-based dressings with layered structures. A representative study by Dhara et al. demonstrated the electrospinning of bilayered structures with different morphologies using polycaprolactone (PCL)-Chitosan emulsions stirred for different durations [75]. Specifically, the emulsions stirred for 5 min produced nanofibers characterized by a porous and loosely organized architecture. Following functionalization with Type I collagen, this layer served as a substrate that mimicked the dermal matrix. In contrast, the emulsions stirred for 12 h generated densely packed nanofibers that structurally replicated the basement membrane, thereby forming an ultrathin yet continuous top layer (Figure 4).

## 4. Functional Chitosan-Based Wound Dressings for Burn Wound Treatment

Second-degree burns damage both the epidermis and part of the dermis, often accompanied by severe pain and a high risk of infection [76,77]. Therefore, it is essential for burn wound dressings to control bacterial growth as well to reduce inflammatory responses [78]. To address this issue, antimicrobial and antioxidant properties have been incorporated into chitosan-based burn wound dressings [79]. For more severe burn wounds, tissue engineering techniques are often required to assist in wound repair. Hence, there is a growing interest in developing new chitosan-based burn wound dressings containing bioactive components to promote tissue regeneration at the wound sites. In this section, we will present an overview of the design and application of functional chitosan-based dressing materials. We will begin by exploring strategies for developing chitosan-based burn wound dressings possessing antimicrobial and/or antioxidant properties, followed by introducing the recent advances in tissue engineering facilitated by chitosan-based dressings.

### 4.1. Antimicrobial and Antioxidant Chitosan-Based Burn Wound Dressings

Despite the antimicrobial nature of chitosan and its derivatives, additional antimicrobial agents are often incorporated to provide a better protection for the burn wounds against infection. To achieve this goal, the most straightforward approach is to direct mixing antimicrobial agents into the chitosan-based burn wound dressing. For example, Doaa Alshora et al. prepared a burn wound dressing in the film form composed of chitosan and sodium alginate and introduced silver sulfadiazine [80], a small-molecular antimicrobial agent, into this burn wound dressing using the solvent casting method. Compared with non-medicated chitosan-sodium alginate film, the silver sulfadiazine -loaded biofilm exhibited significantly superior antibacterial effects [81].

**Figure 5:**
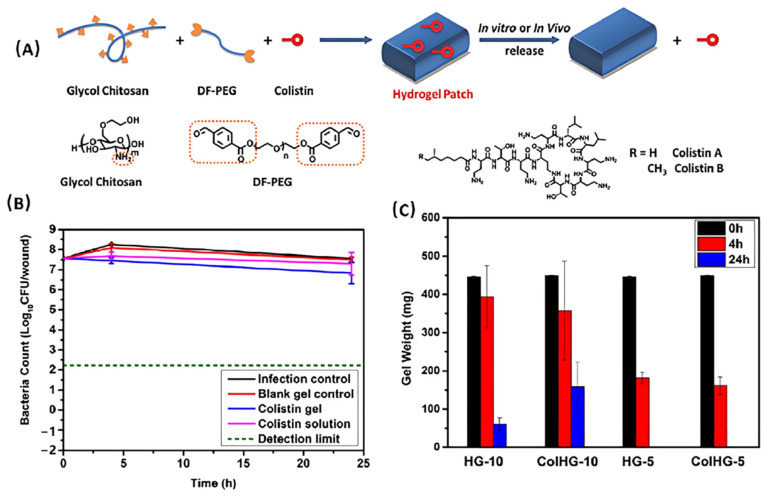
(**A**) Schematic diagram of the synthesis of the colistin-containing hydrogel. (**B**) The animal “burn” infection model test of the colistin-loaded hydrogel against colistin-resistant strain at a low dose (0.3 mg/wound). Black line: blank infection control; red line: blank HG-10 hydrogel; blue line: HG 10 with colistin; pink line: colistin solution. The detection limit is shown in dash line. (**C**) The weight loss of the colistin-loaded hydrogels in vivo over the time. Adapted with permission from Ref. [83]. Copyright © 2016 The WILEY-VCH (Weinheim, Germany).

Antimicrobial agents can also be encapsulated into chitosan-based hydrogels in addition to simple mixing [82]. For instance, colistin is a potent polypeptide antibiotic against various Gram-negative “superbugs” such as Pseudomonas aeruginosa. However, its potential nephrotoxicity limits its application. To tackle this problem, Velkov and Haddleton et al. integrated colistin into a glycol chitosan-based hydrogel by forming imine bonds among colistin, glycol chitosan, and an aldehyde-modified poly (ethylene glycol) crosslinker (DF-PEG) [83], as illustrated in Figure 5A. Within this system, colistin was uniformly distributed in the hydrogel, leading to a high drug loading efficiency. More interestingly, the presence of colistin also accelerated the gelation process, indicating the incorporation of colistin is beyond simple encapsulation. In an in vivo burn infection model, the colistin-loaded hydrogel not only killed colistin-sensitive Pseudomonas aeruginosa strains effectively, but also demonstrated potency against colistin-resistant “superbugs”. (Figure 5B,C). Owing to the dynamic feature of imine bonds as well as the biodegradability of glycol chitosan, this burn wound dressing showed “on-wound” degradation over 24 h.

In addition to infections, when a burn occurs, the skin and surrounding tissues are severely damaged, immediately triggering a series of complex inflammatory responses [84]. During this process, immune cells respond rapidly, releasing a large number of inflammatory mediators, including reactive oxygen species (ROS) and reactive nitrogen species (RNS), which play crucial roles in clearing pathogens and damaged cells. However, excessive ROS and RNS can also lead to oxidative stress, further exacerbating tissue damage and causing severe complications such as systemic inflammatory response syndrome, immunosuppression, infection, sepsis, and multiple organ failure.

Incorporating antioxidants into chitosan-based burn wound dressings is an effective approach to address the abovementioned challenge. Antioxidants can be categorized into non-enzymatic and enzymatic types based on their mechanisms of action [85]. Non-enzymatic antioxidants include low-molecular-weight compounds such as vitamin E, vitamin C, flavonoids, and glutathione, while enzymatic antioxidants include catalase and superoxide dismutase. Additionally, some natural products, such as curcumin, salvianolic acid, crocin, and quercetin, also show strong antioxidative activity. They can protect burn wounds from oxidative stress damage through various mechanisms, such as scavenging free radicals, inhibiting oxidase activity, and regulating the expression of antioxidant enzymes. Through a similar encapsulation strategy as described in antimicrobial burn wound dressing, chitosan-based dressings can also be loaded with antioxidants to further enhance their antioxidant properties.

For example, Salvianolic acid B is an active component extracted from the traditional Chinese herb Salvia miltiorrhiza, known for its excellent antioxidant and anti-inflammatory properties. Incorporating Salvianolic acid B, He and Zhang et al. designed an antioxidant hydrogel dressing for burn treatment. Crosslinked through the dynamic imine bonds between glycol chitosan and tetra-arm polyethylene glycol (terminated with acetaldehyde or benzaldehyde) [1], this hydrogel exhibits shape adaptability, self-healing property, as well as rapid degradation. The encapsulated Salvianolic acid B, on the other hand, can be released to the wound site through a combined mechanism of passive diffusion and hydrogel degradation, showing excellent antioxidant performance in vitro. In a rat model of deep second-degree burn wounds, the Salvianolic acid B-loaded hydrogel rapidly reduced wound temperature, modulated the oxidative microenvironment of the wound, promoted angiogenesis, and eventually accelerated wound healing.

Recently, it has been found that the presence of bacteria often induces oxidative stress in wounds, while the wounds under oxidative stress are more prone to bacterial colonization [86]. Therefore, the combination of antibacterial and antioxidant properties can further enhance the therapeutic effect of dressings on burn wounds. For example, baicalein, which exhibits excellent antimicrobial and antioxidant activities, was used by Wang, Bai, Du, and their research team to develop a dual-functional nanofiber membrane for burn wound treatment [87]. In this study, baicalein was first modified using valeric anhydride and then reacted with the amino groups in chitosan to form baicalein-modified chitosan. This baicalein-modified chitosan was subsequently combined with polyvinyl alcohol and electrospun into a nanofiber membrane. In vitro experiments demonstrated that the baicalein-modified chitosan nanofiber membrane not only effectively neutralizes ROS but also significantly inhibits the growth of both Staphylococcus aureus and Escherichia coli. Furthermore, the nanofiber membrane exhibited outstanding antimicrobial and wound-healing effects in infected wound models.

**Figure 6:**
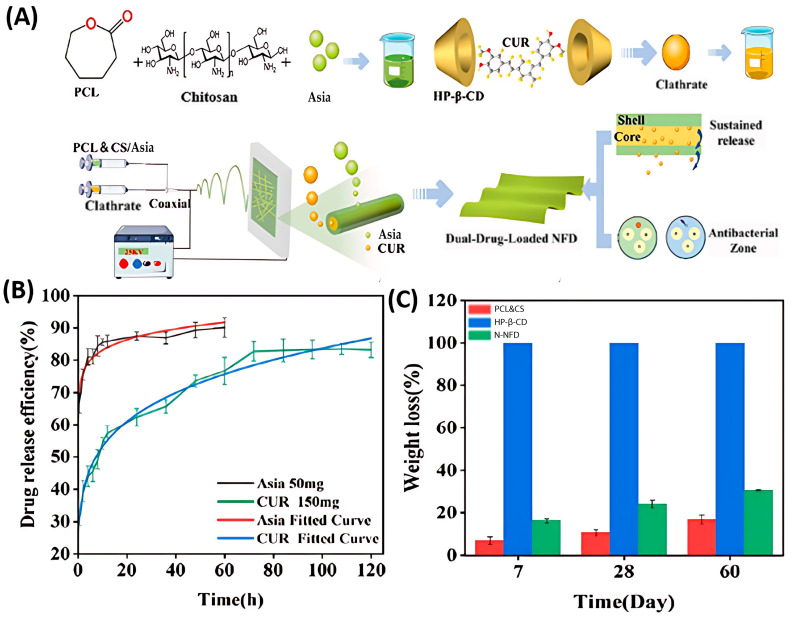
(**A**) Preparation process of the dual-loaded NFD; (**B**) Release curves of Asia and CUR; (**C**) Statistical graph of the degradation of N-NFD, PCL&CS and HP-β-CD at 7, 28 and 60 days in PBS solution. Adapted with permission from Ref. [88]. Copyright © 2024, American Chemical Society (Washington, DC, USA).

Apart from encapsulating a single active ingredient, chitosan-based dressings that are loaded with multiple components have the potential to deliver programmable antimicrobial and antioxidant effects, providing an enhanced therapeutic strategy to address the varied requirements of wound healing throughout its different stages. For instance, Nie, Ma, and their team prepared a core-shell structured dual-drug-loaded nanofiber dressing using electrospinning technology for the treatment of deep second-degree burns [88]. The shell, composed of Polycaprolactone and Chitosan, was loaded with Asiaticoside (Asia) to promote collagen deposition and tissue repair. The core, on the other side, encapsulated curcumin using 2-hydroxypropyl-β-cyclodextrin (HP-β-CD) for sustained release (Figure 6A). Chitosan in the shell provided biocompatibility and antimicrobial properties, while the porous structure in the nanofiber dressing ensured uniform drug distribution and release. In vitro, 66.82% of Asia was rapidly released within 0.5 h, while 83.22% of CUR was released over 120 h (Figure 6B). Mass loss ratios of the nanofiber components in PBS buffer confirmed these release rates (Figure 6C). In a rat model, the dressing achieved a 95% wound healing rate within 14 days, with significant collagen deposition and angiogenesis.

### 4.2. Chitosan-Based Burn Wound Dressings for Tissue Engineering

In addition to providing antibacterial activity and an antioxidant microenvironment, the treatment of deep partial-thickness burns wounds or third-degree burn wounds often requires the integration of tissue engineering strategies [89]. With the aid of bioactive scaffolds and suitable bioactive additives, tissue engineering to activate critical repair mechanisms such as cell migration, angiogenesis, and tissue regeneration. In recent years, chi-tosan-based dressings have shown power in tissue engineering [90]. Owing to the extracellular matrix-mimicking structure of chitosan, chitosan-based dressings are known to promote cellular adhesion and proliferation. Furthermore, by incorporating suitable bioactive components (such as growth factors, genetic materials, stem cells), chitosan-based dressings can be transformed into versatile platforms for treating different burn wounds.

**Figure 7:**
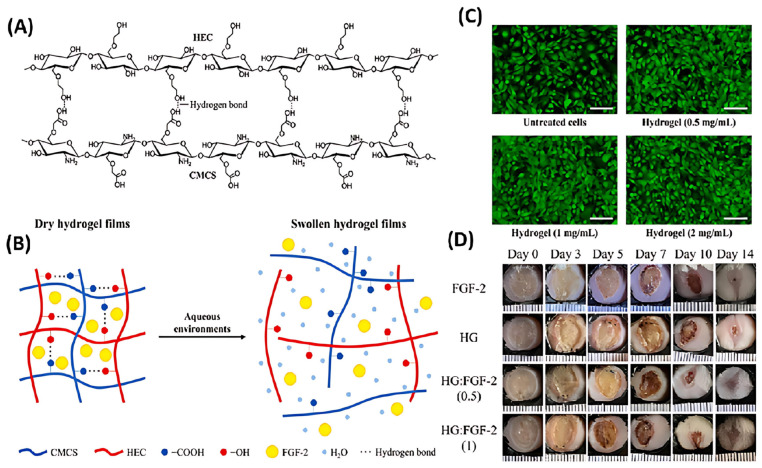
(**A**) Interpolymeric bonding between CMCS and HEC. (**B**) Release of FGF-2 from the swollen hydrogel film in an aqueous environment. (**C**) Effects of hydrogel of different concentrations in the culture medium on the proliferation of NIH/3T3 cells. (**D**) Optical photographs of burn wounds treated with hydrogel films with different FGF-2 release amounts. Adapted with permission from Ref. [91]. Copyright © 2023 Elsevier B.V. (Amsterdam, The Netherlands).

Growth factor therapy delivers growth factors to wound sites, activating the proliferation of surrounding cells to facilitate wound healing. Chitosan-based dressings not only serve as a delivery system for growth factors but also promote tissue regeneration through their inherent biological activity. For instance, Hieu Tran-Van et al. developed a hydrogel film composed of carboxymethyl chitosan (CMCS) and hydroxyethyl cellulose (HEC) for the delivery of fibroblast growth factor (FGF-2), accelerating the repair of burn wounds [91]. This hydrogel film is formed through hydrogen bonding between CMCS and HEC, creating an interpenetrating polymer network with excellent swelling properties and controlled drug release capabilities, while effectively avoiding the potential toxicity issues associated with chemical cross-linking agents (Figure 7A,B). Studies have shown that the CMCS/HEC hydrogel film significantly promotes the proliferation of NIH/3T3 fibroblasts (Figure 7C), protects FGF-2 from protease degradation in vitro, and exhibits remarkable wound healing effects in a burn mouse model (Figure 7D), including accelerated epithelialization, enhanced formation of granulation tissue and blood vessels, and reduced scar formation.

Gene therapy involves delivering genetic material (DNA/RNA) into cells using viral or non-viral vectors to restore or correct cellular functions. Chitosan, positively charged at physiological pH, binds to negatively charged DNA/RNA, forming protective nanoparticles that resist enzymatic degradation. As a result, chitosan-based materials are extensively studied as non-viral gene delivery vectors. However, bio-derived chitosan suffers from poor water solubility and low transfection efficiency. To address these issues, researchers have chemically modified chitosan by incorporating basic amino acids like arginine, histidine, and lysine, enhancing its water solubility and mimicking viral envelope components [92]. Notably, arginine-modified chitosan (Arg-CS) demonstrates high gene transfection efficiency both in vitro and in vivo, showcasing its potential for gene therapy applications.

**Figure 8:**
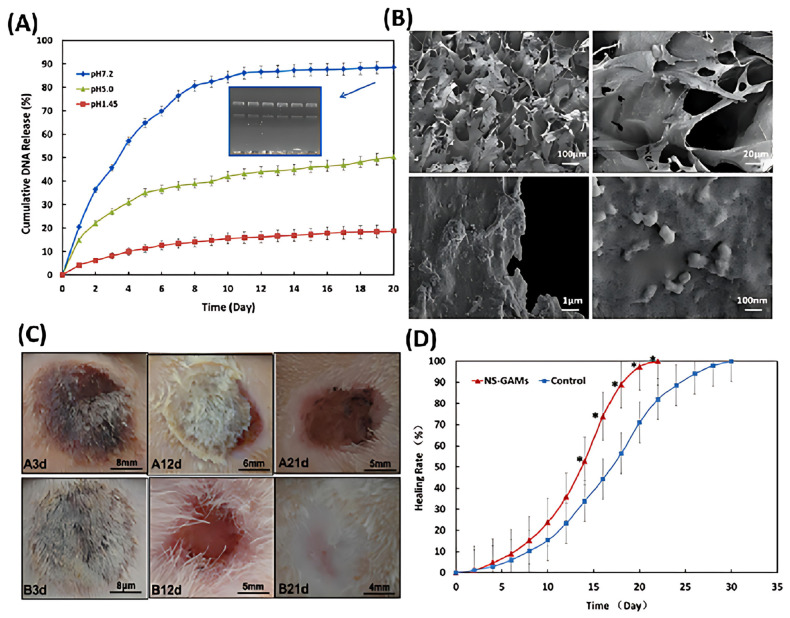
In vitro release of pDNA from NS-GAM. (**A**) Cumulative amount of pDNA released in vitro from NS-GAM and agarose gel electrophoresis of plasmids. (**B**) SEM of the surface of NS-GAM. Gross examination and healing rate. (**C**) Observation of deep 2nd degree burn wound. upper: control group; Below: NS-GAM group. (**D**) The calculated wound size decreases. Adapted with permission from Ref. [93]. Copy © 2021 Elsevier B.V. (Amsterdam, The Netherlands).

Inspired by this, Chang et al. utilized Arg-CS as a gene delivery vector and developed a composite hydrogel as gene-activated matrix (GAM) for in situ treatment of deep second-degree burn wounds [93]. They first produced therapeutic plasmids/Arg-CS complexes by complexing Arg-CS with plasmid DNA (pDNA) encoding mVEGF165 and TGF-β1. These complexes were then incorporated into a composite hydrogel based on N-carboxymethyl chitosan and sodium alginate (NS-GAM). This hydrogel, with an average pore size of 100 μm and a porosity of 50.9%, facilitated pDNA release (Figure 8A,B) and supported cell adhesion and growth. In vitro, the hydrogel loaded with gene fragments sustained gene expression for at least 9 days and protected the gene fragments from enzymatic degradation and immune responses during delivery. In a rat burn model, the experimental group treated with this burn wound dressing achieved complete wound healing within 22 days (Figure 8C,D), with significantly increased expression levels of VEGF and TGF-β1 proteins, promoting neovascularization and collagen regeneration.

**Figure 9:**
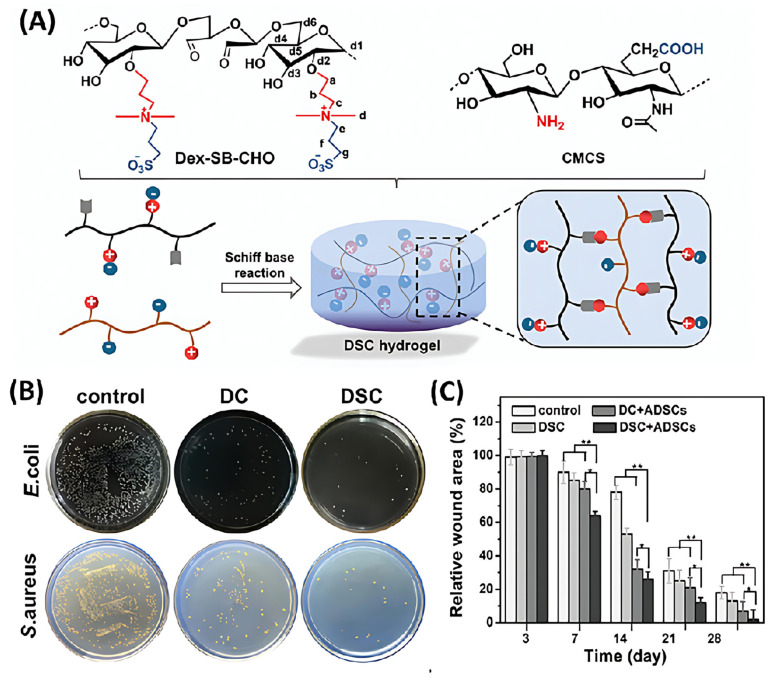
(**A**) Preparation of zwitterionic polysaccharide hydrogel. (**B**) Optical photographs of the relative adsorption of proteins on the surface of DC hydrogel (without zwitterionic modification) and DSC hydrogel. (**C**) The relative wound closure area, all experiments were performed in triplicate and data are reported as mean ± SD (n = 3), ** *p* < 0.01. Adapted with permission from Ref. [4]. Copy © 2022 Wiley-VCH GmbH (Weinheim, Germany).

In addition to gene therapy, stem cell therapy has also garnered considerable attention in burn wound treatment in recent years. Unlike growth factor or gene therapy, which promotes the cell growth around the wound, stem cell therapy leverages the self-renewal and differentiation of stem cells to direct participate in the remodeling of burn-injured tissues. Combining stem cells with chitosan-based dressings can serve as a carrier material for tissue engineering, providing a suitable microenvironment for the delivery and cultivation of stem cells. For example, Yao and Li et al. developed a zwitterionic polysaccharide hydrogel based on chitosan [4] (Figure 9A). Formed by the dynamic imine bonds between sulfobetaine-modified dextran and carboxymethyl chitosan (Dex-SB-CHO), this hydrogel was injectable with self-healing performance, allowing the delivery of adipose-derived stem cells (ADSCs) to the deep wound site. Moreover, the hydrogel exhibited high nonfouling and antimicrobial properties, creating a clean microenvironment for proliferation and maintaining the stemness of ADSCs. (Figure 9B). Furthermore, this hydrogel evaded immune system recognition, reducing inflammation, thereby promoting collagen deposition as well as angiogenesis. In a mouse burn model, the ADSC-loaded hydrogel minimized the wound area in 28 days (Figure 9C) and offered scarless skin tissue regeneration.

## 5. Outlook and Summary

### 5.1. Outlook

Chitosan and its derivatives have a broader range of raw material sources than other biomacromolecules [94], ranging from traditional crustaceans and fungi to emerging sources such as bee and silkworm exoskeletons [95]. However, this diversity leads to quality variability, posing challenges for medical applications. Variations in molecular weight, degree of deacetylation, processing methods [96], and batch-to-batch differences in chain length and crystallinity result in inconsistent performance [97], while residual impurities may also elevate immunogenicity risks [98]. Furthermore, the lack of standardized detection methods causes discrepancies in the measurement of critical parameters. For instance, when the degree of deacetylation of the same batch of chitosan was tested by infrared spectroscopy versus titration method, the results differed significantly, rendering the data incomparable, further complicating standardization and regulatory [91]. To facilitate clinical use, it is essential to establish unified quality standards, optimize production processes, develop standardized testing methods and material traceability procedures. Concurrently, regulatory oversight should extend beyond chitosan raw materials to encompass various forms of chitosan-based dressing products, alongside the formulation of product-specific regulations. Such as standards for hydrogel water content and permeability, or specifications for nanofiber diameters in burn wound dressings.

Additionally, there is still potential for improvement in the management of burn wound exudate using chitosan-based dressings [99]. At present, these dressings primarily rely on their hydrophilic and porous structures to absorb exudate, which may not be sufficient when dealing with large volumes of exudate [100]. Janus dressings with asymmetric structures and unidirectional biofluid transport properties present a novel strategy for tackling this challenge [101]. Through the combination of advanced techniques like electrospinning and 3D printing, coupled with thoughtful molecular engineering and structural refinement, it is expected that chitosan-based burn wound dressings with unidirectional biofluid transport will prevent exudate reflux and secondary injuries resulting from dressing adherence. Additionally, the incorporation of intelligent monitoring components into chitosan materials paves the way for “smart” wound management [6]. By embedding sensors into these dressings may facilitate the continuous collection and analysis of burn exudate parameters, offering real-time insights to track wound healing progress and assess treatment effectiveness.

Furthermore, to address the complex and dynamic microenvironments encountered during the various stages of burn wound healing, future research should focus on developing chitosan-based burn wound dressings that offer multifunctionality and high tunability. Specifically, drawing on recent advancements in multi-component reactions for polymer modification [102], chemically modified chitosan through these reactions to produce multifunctional burn wound dressings are expected to enhance the efficacy of burn treatment. Simultaneously, integrating highly biocompatible responsive motifs, such as enzyme-responsive modules, into existing chitosan-based burn wound dressings enables them to dynamically respond to changes in the wound microenvironment throughout the healing process, thereby facilitating more efficient wound care. Moreover, it is important to develop chitosan-based burn wound dressings with enhanced biomimetic performance. These dressings should replicate the multilayered structure, mechanical properties, and biological functions of human skin, thereby offering superior protection, supporting tissue regeneration, and effectively minimizing scar formation. In summary, by conducting more in-depth research on chitosan dressings, we can offer personalized and adaptive solutions for burn treatment, tailored to the unique needs of each patient and the specific stages of the healing process.

### 5.2. Summary

Chitosan-based dressings have emerged as promising biomaterials for burn wound care due to their inherent biocompatibility, antimicrobial activity, and tunable functionalities. In this mini-review, we summarize recent advancements in this field, with a focus on hydrogel and micro/nanofiber formulations. These dressings not only act as physical barriers to protect wound surfaces but also maintain a moist environment conducive to healing. Crucially, they can be tailored to modulate the wound microenvironment based on burn severity, while simultaneously serving as scaffolds to support tissue regeneration in severe injuries. Despite substantial evidence supporting their therapeutic potential, the clinical translation of chitosan dressings faces hurdles such as regulatory approvals and scalable manufacturing. Continuous refinement of fabrication processes, develop standardized testing methods as well to adjust mechanical robustness and functions, is expected to overcome limitations of conventional burn wound dressings, thereby providing more efficient and patient-friendly solutions for burn treatment. Furthermore, the development of next-generation chitosan-based materials with stimuli-responsive and multifunctional capabilities holds promise for advancing wound management and improving rehabilitation outcomes for burn patients.

## Figures and Tables

**Table 1:** The common approaches to chemically modify chitosan for improved solubility.

Chemically Modified Chitosan	Principle of Design	Reagents for Modification	Chemical Formula	Reference
Carboxymethyl chitosan	Introduce hydrophilic groups into chitosan	Chloroacetic acid or its sodium salt	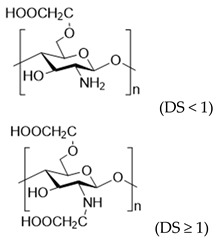	[39]
Quaternary amino chitosan		Quaternizing agents, such as methyl iodide and glycidyl trimethylammonium chloride	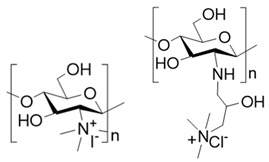	[40,41]
Acylated chitosan	Disrupt the intramolecular and intermolecular hydrogen bonds of chitosan	Organic acids, acid anhydrides or acyl chlorides	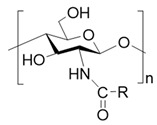	[42]
Alkylated chitosan		Halogenated hydrocarbons or higher aliphatic aldehydes	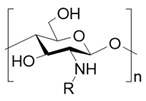	[43,44,45]

## Data Availability

No new data were created.
